# Supplementary material for: Enhanced immunocompatibility of ligand-targeted liposomes by attenuating natural IgM absorption
Source: Nat Commun. 2018 Jul 30;9:2982. doi: 10.1038/s41467-018-05384-1 (PMC6065320; doi:10.1038/s41467-018-05384-1)
Supplement: Supplementary file 1 — Supplementary Information [file 41467_2018_5384_MOESM1_ESM.pdf]

Supplementary Information

Enhanced Immunocompatibility of Ligand-targeted Liposomes by Attenuating Natural IgM Absorption

Guan and Shen et al

Supplementary Figures

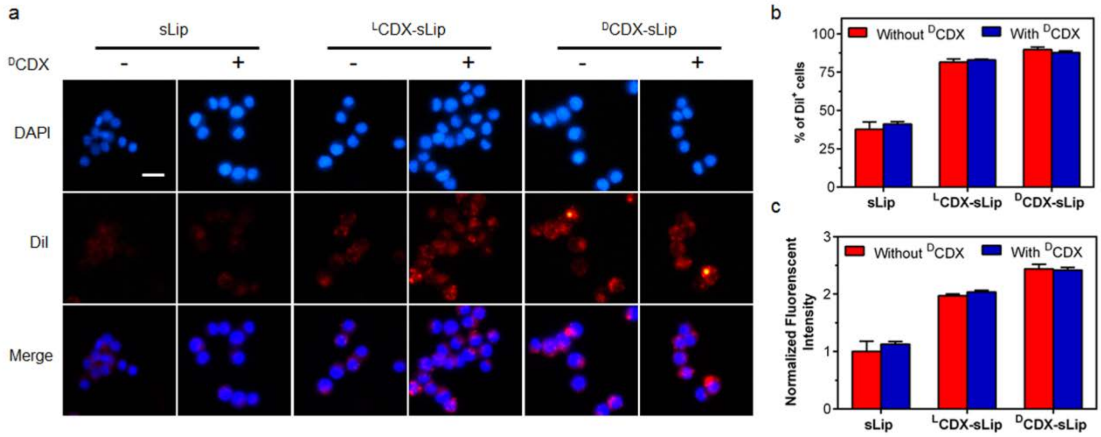

Supplementary Figure 1. The uptake of sLip, ^{L}CDX-sLip and ^{D}CDX-sLip by RAW264.7 cells. (a) Microscopic observation and (b, c) quantitative analysis of phagocytosis of sLip, ^{L}CDX-sLip and ^{D}CDX-sLip with or without 2 h pre-incubation with 200 μM free ^{D}CDX peptide. Scale bar = 20 μm. n = 3, Data are means ± s.d.

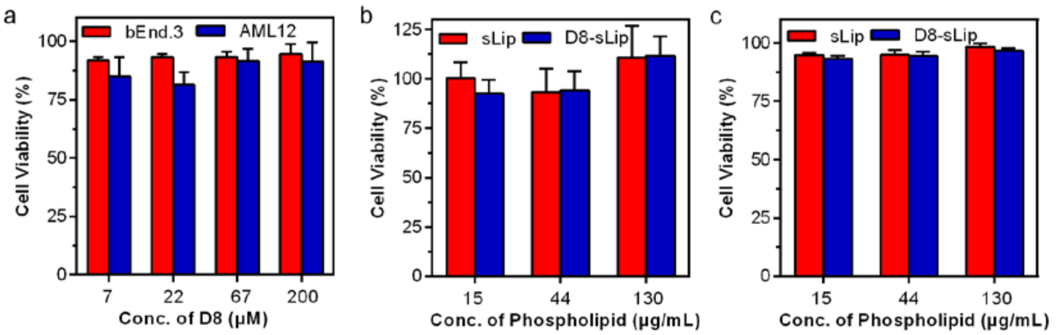

Supplementary Figure 2. Cytotoxicity of free D8 peptide and D8-sLip. (a) Cytotoxicity of free D8 peptide against bEnd.3 and AML12 cells. sLip and D8-sLip against (b) AML12 and (c) bEnd.3 cells after 48 h incubation at 37 °C. Data are means ± s.d. (n = 3).

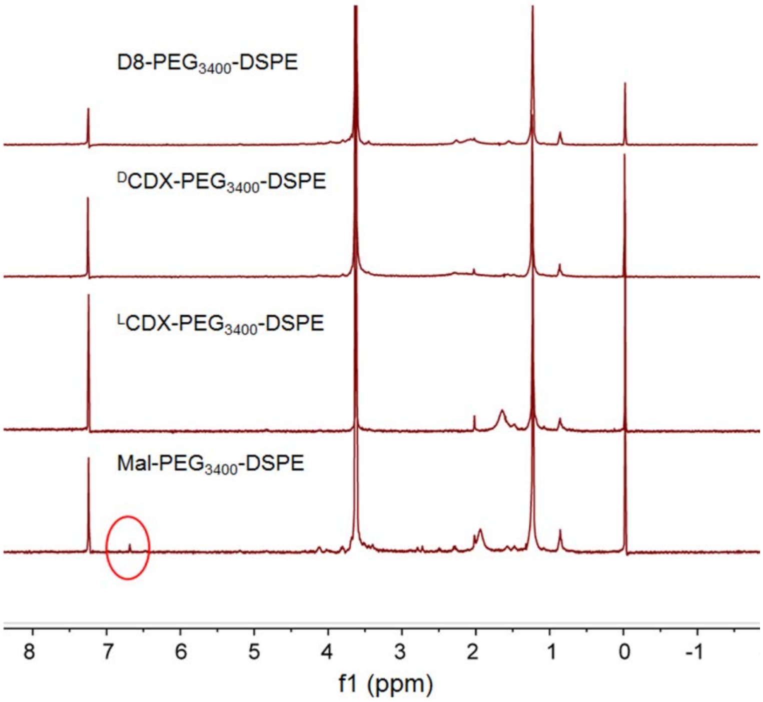

Supplementary Figure 3. 1H-NMR spectra of mal-PEG3400-DSPE and peptide-PEG3400-DSPE. The characteristic peak of maleimide at 6.8 ppm was circled.

Supplementary Tables

Supplementary Table 1. Area under the curve (AUC_{0-24}) of liposomes (50 mg HSPC per kg of mouse) with or without pre-dose of blank liposomes (5 mg HSPC per kg of mouse, no DiI loading) in BALB/c mice. n=3, data are means ± s.d.

	AUC_{0-24} (μg·h mL^{-1})	
	Without pre-dose	With pre-dose
sLip	584.2 ± 6.8	350.3 ± 10.7
^{L}CDX-sLip	164.8 ± 10.1	8.7 ± 4.7
^{D}CDX-sLip	18.3 ± 2.4	7.3 ± 0.6
D8-sLip	286.8 ± 21.5	112.7 ± 13.9

Supplementary Table 2. Characterization of sLip, ^{L}CDX-sLip and ^{D}CDX-sLip with or without pre-incubation with serum at 37°C for 1 h, n = 3, data are means ± s.d.

	Without serum			With serum		
	Size (nm)	PDI	Zeta (mV)	Size (nm)	PDI	Zeta (mV)
sLip	138.9±0.72	0.059±0.02	-48.33±1.97	120.8±0.98	0.286±0.01	-19.23±1.57
^{L}CDX-sLip	138.8±0.14	0.086±0.02	-30.87±0.95	123.4±2.82	0.246±0.02	-14.63±1.28
^{D}CDX-sLip	136.4±2.48	0.057±0.01	-31.45±0.46	118.8±1.56	0.301±0.02	-15.46±2.02

Supplementary Table 3. Peptide sequences.

Peptide	Sequence	Length (AA)
^{L}CDX	FKESWREARGTRIERG	16
^{D}CDX	G^{D}R^{D}E^{D}I^{D}R^{D}TG^{D}R^{D}A^{D}E^{D}R^{D}W^{D}S^{D}E^{D}K^{D}F	16
^{D}CDX-1	G^{D}R^{D}A^{D}I^{D}R^{D}TG^{D}R^{D}A^{D}E^{D}R^{D}W^{D}S^{D}A^{D}K^{D}F	16
^{D}CDX-2	G^{D}A^{D}E^{D}I^{D}R^{D}TG^{D}R^{D}A^{D}E^{D}R^{D}W^{D}S^{D}E^{D}A^{D}F	16
^{D}CDX-S8	^{D}R^{D}TG^{D}R^{D}A^{D}E^{D}R^{D}W	8
ANG	TFFYGGSRGKRNNFKTEEY	19
D8	^{D}RTG^{D}R^{D}A^{D}RE^{D}W	8

Supplementary Table 4. Characterization of different liposomes shown in the table with or without pre-incubation of serum at 37°C for 1 h. Size and zeta potential were all measured by Malvern laser particle size analyzer. n = 3, data are means ± s.d.

Liposomes	Without serum			With serum		
	Size (nm)	PDI	Zeta-potential (mV)	Size (nm)	PDI	Zeta-potential (mV)
D8-sLip	135.7±1.79	0.017±0.02	-38.5±1.73	128.6±1.28	0.302±0.01	-17.2±1.02
DOTAP-sLip	157.8±0.57	0.104±0.02	59.5±0.95	122.5±0.56	0.247±0.02	-16.9±0.86
^{D}CDX-1-sLip	141.6±0.46	0.116±0.04	17.5±2.96	125.8±0.58	0.267±0.01	-20.4±1.31
^{D}CDX-2-sLip	136.9±1.34	0.163±0.02	-38.9±0.89	99.74±0.86	0.222±0.03	-19.4±0.97
Angiopep-2-sLip	128.1±1.90	0.091±0.02	-32.6±1.69	246.9±1.97	0.683±0.02	-17.1±0.58
^{D}CDX-S8-sLip	136.5±2.19	0.064±0.01	-37.9±0.20	124.6±1.86	0.158±0.03	-16.1±0.75

Supplementary Table 5. Predicted free energies of binding of the peptides with α7 nAChR

Peptide sequence	Predicted ΔG [kcal mol^{-1}]
$G^{D}R^{D}E^{D}I^{D}R^{D}TG^{D}R^{D}A^{D}E^{D}R^{D}W^{D}S^{D}E^{D}K^{D}F$	-10.11
$^{D}R^{D}TG^{D}R^{D}A^{D}E^{D}R^{D}W$	-10.47
$^{D}R^{D}TG^{D}R^{D}A^{D}RE^{D}W$	-11.06
$^{D}RTG^{D}R^{D}A^{D}RE^{D}W$	-11.48
